# A Machine Learning Approach to Support Urgent Stroke Triage Using Administrative Data and Social Determinants of Health at Hospital Presentation: Retrospective Study

**DOI:** 10.2196/36477

**Published:** 2023-01-30

**Authors:** Min Chen, Xuan Tan, Rema Padman

**Affiliations:** 1 Department of Information Systems & Business Analytics College of Business Florida International University Miami, FL United States; 2 Department of Information Systems and Analytics Leavey School of Business Santa Clara University Santa Clara, CA United States; 3 The H John Heinz III College of Information Systems and Public Policy Carnegie Mellon University Pittsburgh, PA United States

**Keywords:** stroke, diagnosis, triage, decision support, social determinants of health, prediction, machine learning, interpretability, medical decision-making, retrospective study, claims data

## Abstract

**Background:**

The key to effective stroke management is timely diagnosis and triage. Machine learning (ML) methods developed to assist in detecting stroke have focused on interpreting detailed clinical data such as clinical notes and diagnostic imaging results. However, such information may not be readily available when patients are initially triaged, particularly in rural and underserved communities.

**Objective:**

This study aimed to develop an ML stroke prediction algorithm based on data widely available at the time of patients’ hospital presentations and assess the added value of social determinants of health (SDoH) in stroke prediction.

**Methods:**

We conducted a retrospective study of the emergency department and hospitalization records from 2012 to 2014 from all the acute care hospitals in the state of Florida, merged with the SDoH data from the American Community Survey. A case-control design was adopted to construct stroke and stroke mimic cohorts. We compared the algorithm performance and feature importance measures of the ML models (ie, gradient boosting machine and random forest) with those of the logistic regression model based on 3 sets of predictors. To provide insights into the prediction and ultimately assist care providers in decision-making, we used TreeSHAP for tree-based ML models to explain the stroke prediction.

**Results:**

Our analysis included 143,203 hospital visits of unique patients, and it was confirmed based on the principal diagnosis at discharge that 73% (n=104,662) of these patients had a stroke. The approach proposed in this study has high sensitivity and is particularly effective at reducing the misdiagnosis of dangerous stroke chameleons (false-negative rate <4%). ML classifiers consistently outperformed the benchmark logistic regression in all 3 input combinations. We found significant consistency across the models in the features that explain their performance. The most important features are age, the number of chronic conditions on admission, and primary payer (eg, Medicare or private insurance). Although both the individual- and community-level SDoH features helped improve the predictive performance of the models, the inclusion of the individual-level SDoH features led to a much larger improvement (area under the receiver operating characteristic curve increased from 0.694 to 0.823) than the inclusion of the community-level SDoH features (area under the receiver operating characteristic curve increased from 0.823 to 0.829).

**Conclusions:**

Using data widely available at the time of patients’ hospital presentations, we developed a stroke prediction model with high sensitivity and reasonable specificity. The prediction algorithm uses variables that are routinely collected by providers and payers and might be useful in underresourced hospitals with limited availability of sensitive diagnostic tools or incomplete data-gathering capabilities.

## Introduction

### Background

Diagnostic errors have emerged as a major public health problem, contributing to preventable patient harm and excess health spending. A recent US National Academies report titled “Improving Diagnosis in Healthcare” suggested that medical misdiagnosis is likely to affect almost everyone at least once in their lifetime, sometimes with devastating consequences [1]. Misdiagnosis accounts for at least 40,000 to 80,000 hospital deaths and probably a comparable amount of disability annually in the United States [2]. Physician-reported errors and closed malpractice claims indicate that stroke is among the most common and dangerous misdiagnosed medical conditions [3-5]. Preventable deaths from stroke due to diagnostic errors occur ≥30 times more often than deaths from myocardial infarction [6,7].

The diagnosis of stroke is complicated by the abundance of stroke mimics and stroke chameleons. Approximately 30% of patients admitted to hospitals with typical stroke symptoms ended up having nonstroke conditions (ie, stroke mimics) [8]. A wide range of other medical conditions can exhibit symptoms that mimic strokes, such as seizures, migraines, psychiatric disorders, and drug or alcohol intoxication [8,9]. Mistaking a mimic for acute stroke may expose patients to unnecessary diagnostics and therapy, waste limited resources, and incur additional costs. Conversely, and more dangerously, stroke chameleons are actual stroke conditions presenting with atypical or underrecognized stroke symptoms and masquerading as nonstroke medical conditions. Approximately 25% of patients who had a stroke do not present with typical “face, arm, speech” symptoms at onset, and it is challenging for emergency medical services to identify stroke in such patients [10]. Misdiagnosis of chameleons can lead to significant delays in identifying and treating patients with actual strokes. Approximately one-third of potentially eligible patients failed to receive alteplase (tissue plasminogen activator), the gold-standard treatment for acute ischemic stroke [10]. This is because of either the lack of available specialists to perform appropriate clinical assessments or delays in the process of referring to health care facilities with the required stroke-handling capabilities [11]. In particular, Black people, Hispanic people, women, older people on Medicare, and people in rural areas are more prone to misdiagnosis and delay in receiving tissue plasminogen activator after having a stroke [10]. Furthermore, it is particularly challenging to accurately diagnose stroke in emergency departments (EDs) because of the time-sensitive and dynamic nature of emergency conditions, the fast-paced environment, frequent interruptions, the prevalence of information gaps, and high workload [12-15]. An automated screening tool that can be seamlessly integrated into the clinical workflow to quickly analyze the available information and suggest a diagnosis of stroke (“Stroke Alert” pop-up) could be very helpful [16].

Machine learning (ML), a crucial branch of artificial intelligence, has the potential to identify hidden insights from a large volume of data and generate predictions on unseen data (ie, test data) by iteratively learning from example inputs (ie, training data). ML problems can generally be divided into 3 main types: classification and regression, which are known as supervised learning, and unsupervised learning, which in the context of ML applications often refers to clustering. In the literature on stroke research, ML algorithms have been applied in different tasks, such as identifying factors associated with future stroke risk [17-19], developing stroke severity measures [20,21], and predicting stroke outcomes [22,23]. To improve diagnosis, researchers have focused on developing (electronic health record [EHR]–based) algorithms to determine stroke subtypes [24-26] and applying deep learning methods to facilitate imaging evaluation [27,28]. The recent advances in phenotyping algorithms and deep learning models have significantly improved the prediction for stroke by using multiple types of EHR data, especially clinical notes and advanced diagnostic tests. However, only a few investigations have focused on the application of diagnostic algorithms using ML in emergency triage when detailed clinical assessments and diagnostic tests are not readily available.

The first brain imaging for most patients with suspected stroke is a noncontrast computed tomography (CT) scan, which is completed within minutes of the arrival of the patient to the ED. However, a noncontrast CT scan is not sufficient to diagnose acute stroke, as the head CT test cannot reveal a hyperacute stroke in most cases, and it has reduced sensitivity for lacunar strokes [29]. More sensitive diagnostic tools such as diffusion-weighted magnetic resonance imaging can show ischemic changes very early. Despite the recent increase in the use of advanced neuroimaging, the use of magnetic resonance imaging to diagnose stroke in ED is still limited, especially when diagnosis is urgently required [30]. Moreover, patients who present to EDs can be susceptible to having information gaps because they are usually acutely ill, report quickly to the hospital at irregular hours, and often go to the ED without their primary physician’s knowledge. These factors make it difficult for the attending emergency physician to obtain all the information (eg, clinical notes, reports, and diagnostic test results) needed for making a timely and accurate diagnosis.

Besides medical risk factors, social determinants of health (SDoH) have been shown to be associated with the risk of stroke and many other diseases [31,32]. SDoH include various community and social factors, such as “conditions in which people are born, grow, work, live, and age” and “the fundamental drivers of these conditions” [33]. According to a widely used population health model, only 20% of an individual’s health is tied to clinical care, which includes access to care and the quality of health care services. The other 80% of an individual’s health is tied to their physical environment, social determinants, and behavioral factors such as exercise or smoking [34,35]. In recent years, the increasing focus on population health has led to efforts to address upstream SDoH factors such as access to healthy food and viable transportation options. There is a substantial body of literature devoted to investigating the correlation between various SDoH factors and stroke risk, which has been well documented [36-39]. However, only a few studies have incorporated SDoH information into their prediction model and explicitly evaluated the added value of SDoH information for stroke diagnosis and triage [40]. There is a call, both in the literature and in the practitioner community, to explicitly evaluate whether and how SDoH data can contribute to improving patient risk stratification and prediction [40,41].

### Goal of This Study

In this study, we aimed to develop an ML stroke prediction algorithm based on data widely available at the time of patients’ hospital presentations and to assess the added value of SDoH in stroke prediction. Because the prediction model does not require clinical notes or diagnostic test results, it might be particularly useful in addressing the misdiagnosis challenges faced when dealing with walk-in patients with stroke with milder and atypical symptoms; in low-volume or nonstroke centers’ EDs, where emergency providers have limited daily exposure to stroke [16]; and in rural areas and small communities where there is limited availability of sensitive diagnostic tools and incomplete or unreliable data-gathering capabilities [3,5]. The model could also be applied in emergency medical services and telemedicine to seamlessly triage patients in real time and alert the provider and care team. In addition, we analyzed the most influential driving features helping the diagnosis of each patient and, specifically, the role of SDoH in prediction. The findings can provide insights into the value of prediction models in this critical setting and ultimately assist emergency care providers in making more informed decisions.

## Methods

### Ethics Approval

The secondary hospital discharge data this study examined was from the Healthcare Cost and Utilization Project State-specific databases, Agency for Healthcare Research and Quality. Healthcare Cost and Utilization Project databases conform to the definition of a limited data set, and review by an institutional review board is not required for use of limited data sets [42].

### Data Sources

Our data were obtained from 2 primary sources. We obtained longitudinal administrative data that contained encounter-level information on inpatient stays and ED visits from hospitals in the state of Florida. The second data source was the American Community Survey (ACS) conducted by the US Census Bureau [43]. The ACS data offered zip code–level SDoH information, such as demographic, social, housing, transportation, and other socioeconomic factors.

### Data Extraction and Synthesis

#### The Stroke and Stroke Mimic Cohorts

We adopted a case-control design, and the initial phase of our approach was to create representative examples for model training and ensure that stroke cases and controls have clear separation. We retrospectively extracted 127,114 hospitalization records from 2012 to 2014 with a principal diagnosis of acute cerebrovascular disease in Florida using the clinical classification tool developed by the Agency for Healthcare Research and Quality [44]. Because we wanted to provide timely prediction of the likelihood of a patient’s condition being stroke at the time of hospital presentation, we restricted attention to those variables that care providers can garner when patients first arrive at the hospital (eg, age, gender, race, admission time, primary payer, the number of chronic conditions on admission, etc). Thus, we excluded additional information that can be acquired only during hospitalization or at discharge (eg, procedures performed, length of stay, and total charges).

The key for a model to accurately predict stroke is to distinguish between stroke and stroke-like conditions (“stroke mimics”). We carefully created a stroke mimic data set to simulate tricky diagnostic decision-making and distinguish between actual stroke events and stroke-like events. Using all the records involving patients with nonstroke conditions to construct a prediction model will result in the inclusion of completely irrelevant cases, such as childbirth and hip replacement, and create a highly unbalanced data set. Hence, we consulted physicians about what conditions may show initial symptoms similar to those of a patient with stroke. On the basis of their suggestions, we obtained a list of conditions using Epocrates, a mobile app that health care providers use at the point of care for clinical reference information [45]. The stroke mimics included in the list were brain tumors, conversion and somatization disorders, Wernicke encephalopathy, seizure and postictal deficits, complicated migraines (hemiplegic migraines and migraines with aura), hypoglycemia, and hypertensive encephalopathy. Next, we searched the medical literature to confirm the validity of the list of stroke mimics and built a crosswalk between each stroke mimic and its corresponding International Classification of Diseases, ninth revision, codes. We then used the crosswalk to extract patients whose reasons for visits were one or more of the stroke mimics but subsequent discharge diagnoses were not stroke.

We pooled the stroke and stroke mimic data sets and retained only the data collected during the first admission of the patients. We performed data deduplication once again after combining stroke data and stroke mimic data because a patient may have been first admitted with stroke and readmitted with a stroke mimic condition and vice versa. If a patient appeared in both data sets, we kept only the first occurrence. Because patients may have returned to the hospital multiple times, providers may have obtained more information about patients who are readmitted. Retaining only the index encounter of the patients ensures that our models predict stroke based solely on the information available at the time of a patient’s initial presentation at the hospital. We obtained data from 2010 to 2014, and hence we have 2 years before 2012 as our “cushion period.” The patients included in the analysis were those with no records in 2010 or 2011. The “confirmed stroke” data set contains all the patients whose hospital discharge records confirmed that they had a stroke; thus, it includes not only patients with typical stroke symptoms but also those with mild and atypical symptoms. The stroke mimic data set includes patients with general presentations similar to those of patients with actual stroke, including patients with a discharge diagnosis of epilepsy, diabetes, alcohol, and drug withdrawal. Multimedia Appendix 1 lists the distribution of the top 20 principal diagnoses in the final analysis data set.

#### Feature Extraction and Selection From SDoH Data

The original SDoH data we extracted from the ACS contained a large number of features. We adopted several methods to reduce noise and dimensionality and avoid overfitting. First, we conducted exploratory data analysis such as the principal component analysis to understand the feature distribution and identify patterns and multicollinearity among features. We then combined domain knowledge and a sparse regression method (least absolute shrinkage and selection operator) to remove irrelevant features and merge highly sparse features.

Overall, 4 categories were constructed from a large set of 431 variables in the ACS data for the 983 zip codes in Florida. These categories represent social, economic, housing, occupation, health insurance, and demographic characteristics referenced in the literature as being associated with stroke-related and cardiovascular health status (Multimedia Appendix 2). For example, low income, low education, and poverty have been shown to result in a higher risk of stroke [31,46,47], and low income and low education have been associated with lower heart health and higher risk of heart failure and death [48,49]. Occupation type and education level have been linked to the risk of heart disease [50]. Health insurance status and type have also been linked to cardiovascular health [51,52]. Together, these interlinked socioeconomic factors determine a person’s overall socioeconomic status and, unsurprisingly, have a relationship with health over time. Some of the ACS variables included in the analysis are direct representations of socioeconomic status (eg, average household income and percentage of the population with at least high school–level education), whereas others serve as proxies (eg, percentage of housing units with no vehicles and percentage of the population with a non-English language spoken at home).

We also performed a Markov blanket feature selection method to determine a minimal subset of relevant features that yields the optimal classification performance [53]. Note that tree-based ML algorithms (eg, random forest [RF]) have a built-in feature selection function and inherently eliminate irrelevant features during model training.

**Figure 1:**
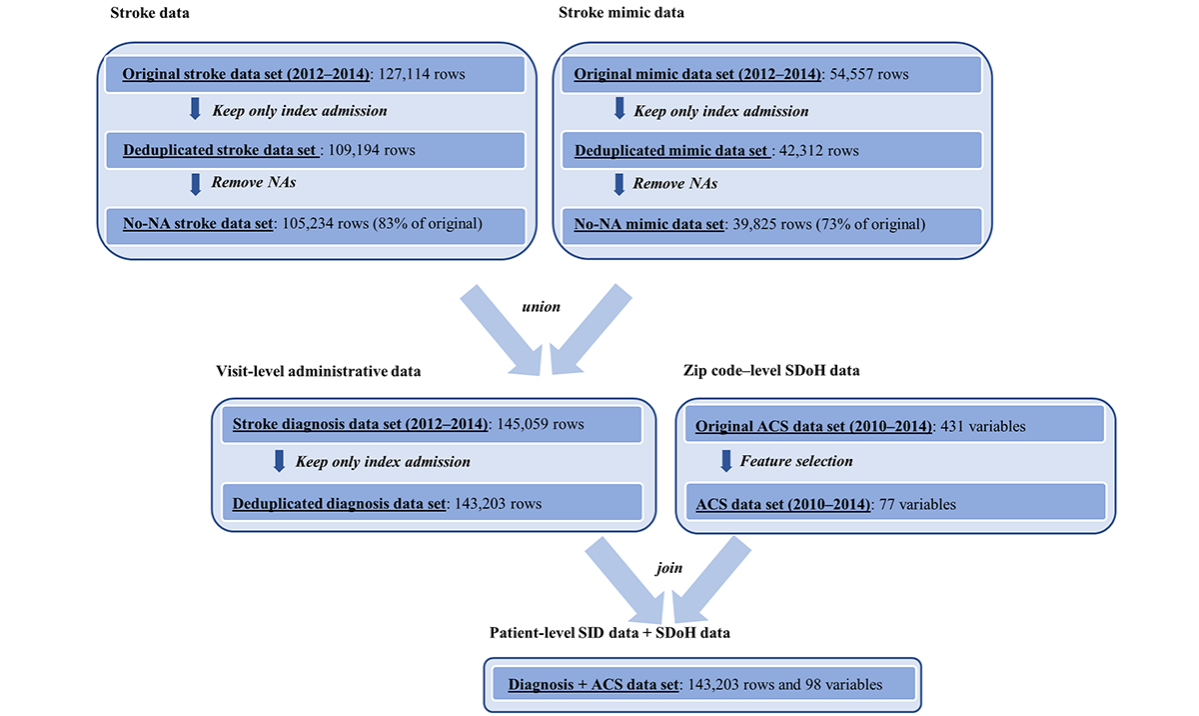
Data processing pipeline. ACS: American Community Survey; NA: not available; SDoH: social determinants of health; SID: State Inpatient Database.

The final analysis data set was formed by merging the patient-level data with the community-level ACS data based on the patients’ zip code information. Figure 1 presents a flowchart of the data processing pipeline. In our final input data set, the number of stroke cases was significantly larger than the number of controls (ie, 73% of the patients were discharged with a confirmed stroke diagnosis, and 27% of the patients ended up having stroke mimics). To address the unbalanced distribution of stroke events in the real-world data, we adopted adaptive synthetic sampling, an oversampling technique for minority class (eg, nonstroke “control”) in the training data [25,54-56].

### Data Modeling and Validation

We started by using the patient-level information available at the time of hospital presentation to predict a binary outcome that indicates whether the patient’s final diagnosis at discharge is stroke. We ran three different models that are well established in the literature for the training process: (1) logistic regression, (2) RF, and (3) gradient boosting machine (GBM). Each model was run with different combinations of predictor variables to assess the added predictive value of the different variables.

Logistic regression is a popular method for modeling the relationship between a set of predictor variables and a binary outcome variable and for benchmarking [57]. RF is a supervised learning algorithm that fits multiple decision trees on different subsamples of data to classify outcomes to prevent the issue of overfitting [58,59]. The predictive accuracy is the average of all the decision trees. It also provides insights into relative feature importance. Parameter tuning helped identify the number of trees and the allowed depth for each tree in the RF that provided the best performance. GBM is similar to RF, as it also constructs multiple decision trees for prediction; however, the difference lies in the way GBM builds the trees and the way it combines the results from the decision trees [60].

We first tuned the hyperparameters of all 3 models to find the optimal configurations using a grid search and 5-fold cross-validation on the entire data set. The evaluation metric used in the cross-validation was the area under the receiver operating characteristic curve (AUC). We used an 80-20 random split on the data set because this is a standard split method used in ML models and is typically performed to test the model performance in designing the ML-enabled diagnostic tool for providers in EDs [16]. We adopted the adaptive synthetic sampling technique to generate synthetic data for the minority class (eg, nonstroke “control”) in the training data to address the unbalanced distribution of stroke events in the real-world data. Using the optimal configurations of hyperparameters, we then developed and assessed our models using a balanced training data set with repeated 5-fold cross-validation and cost-sensitive classification to avoid overfitting. For each fold, the models were evaluated on the performance metrics, including AUC, accuracy, precision, sensitivity or recall, specificity, and *F*_1_-score, using the test data set. The logistic regression and RF models were implemented in Python (version 3.9.12, Python Software Foundation) using scikit-learn (version 1.0.2; David Cournapeau). GBM was implemented in Python 3.9.12 using CatBoost (version 1.0.6, Yandex LLC). The configurations of the key hyperparameters for each of our ML models are listed in Multimedia Appendix 3.

As a robustness check, we adopted an alternative data split method by using historical 2012 data to predict for 2013 and using both 2012 and 2013 data to predict for 2014.

**Figure 2:**
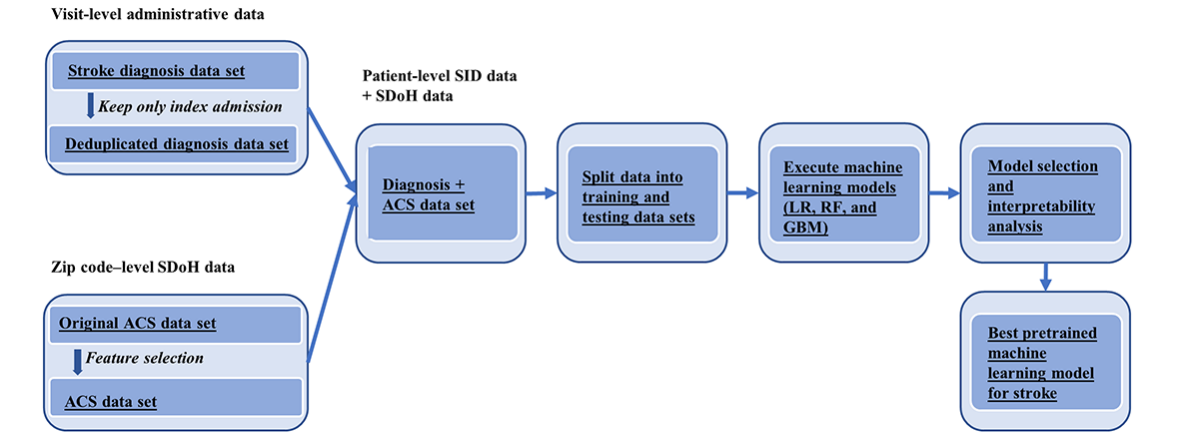
Analysis pipeline. ACS: American Community Survey; GBM: gradient boosting machine; LR: logistic regression; RF: random forest; SDoH: social determinants of health; SID: State Inpatient Database.

Although ML models can produce accurate predictions, they are often treated as black-box models that lack interpretability. This is an important problem, especially in medical care because clinicians are often unwilling to accept machine recommendations without clarity regarding the underlying reasoning [57]. However, according to a recent review, the number of ML studies in the medical domain that addressed explainability is very limited [58]. In this study, we followed the approach outlined by Saarela and Jauhiainen in their 2021 paper [59] to conduct a comparison of feature importance measures to enhance the interpretability or explainability of our models’ results. To provide insights into prediction and ultimately assist care providers in decision-making, we used TreeSHAP for tree-based ML models to explain the stroke prediction for each patient (refer to the details provided in the *Results* section). Figure 2 demonstrates the investigation path followed to develop our models based on the synthesized data and compare and interpret the models to derive the best pretrained ML model for stroke prediction.

## Results

### Descriptive Statistics of the Data Set

In the final data set, there were 143,203 hospital visits of unique patients, and it was confirmed based on the hospital discharge records that 73% (n=104,662) of them had a stroke. The prediction models included 12 patient-level features from the hospital administrative data set, joined by 16 community-level features from the ACS data set. We summarized the patient-level predictors into 3 categories: patient demographics, visit-level features, and individual-level SDoH; their summary statistics are presented in the following table (Table 1). Patients who ended up being diagnosed with stroke tended to be older, have more chronic conditions, and have Medicare as the primary payer. The results of 2-sample *t* tests (2-tailed) with Bonferroni correction showed that all the patient-level predictors were statistically different at a significance level of .05 between the patients with stroke and those with stroke mimics (indicated by the *P* values in the last column of Table 1). The 16 community-level SDoH features were summarized into categories: area demographics, socioeconomic status, occupation, and health insurance coverage at the population level. Multimedia Appendix 2 contains detailed information on the community-level predictors.

**Table 1:** Descriptive statistics of the patient-level predictors.

Features			Total sample (n=143,203), mean (SD)	Stroke cohort (n=104,662), mean (SD)	Stroke mimic cohort (n=38,541), mean (SD)	*P* value
**Patient demographics**						
	Age (years)		65.2843 (19.97)	71.1259 (14.68)	49.4207 (23.49)	<.001
	Sex (female)		0.5019 (0.50)	0.5014 (0.50)	0.5031 (0.50)	.03
	Number of chronic conditions		6.5066 (3.21)	7.1200 (3.00)	4.8410 (3.17)	<.001
	**Race and ethnicity**					
		White	0.6594 (0.47)	0.6736 (0.47)	0.6209 (0.49)	<.001
		Black	0.1802 (0.38)	0.1706 (0.38)	0.2064 (0.40)	<.001
		Hispanic	0.1348 (0.34)	0.1302 (0.34)	0.1472 (0.35)	<.001
		Other races	0.0256 (0.16)	0.0257 (0.16)	0.0255 (0.16)	.04
**Visit-level features**						
	Emergency admission		0.9030 (0.30)	0.9094 (0.29)	0.8859 (0.32)	<.001
	Elective admission		0.0403 (0.20)	0.0214 (0.14)	0.0914 (0.29)	<.001
	Transfer in indicator		0.0913 (0.37)	0.0929 (0.37)	0.0869 (0.36)	<.001
	Night shift^a^		0.3409 (0.47)	0.3257 (0.47)	0.3821 (0.49)	<.001
	Weekend indicator		0.2558 (0.44)	0.2581 (0.44)	0.2496 (0.43)	<.001
**Individual-level SDoH^b^**						
	Urban residence		0.9529 (0.21)	0.9515 (0.21)	0.9567 (0.20)	<.001
	**Primary payer**					
		Medicare	0.6239 (0.48)	0.7027 (0.46)	0.4099 (0.49)	<.001
		Medicaid	0.1103 (0.31)	0.0714 (0.26)	0.2159 (0.41)	<.001
		Private insurance	0.1505 (0.36)	0.1331 (0.34)	0.1980 (0.40)	<.001
		Other payers	0.1153 (0.32)	0.0929 (0.29)	0.1762 (0.38)	<.001
	**Median household income**					
		0-25th percentile	0.4025 (0.49)	0.3984 (0.49)	0.4134 (0.49)	<.001
		26th-50th percentile	0.3261 (0.47)	0.3289 (0.47)	0.3186 (0.47)	<.001
		51st-75th percentile	0.1992 (0.40)	0.1994 (0.40)	0.1986 (0.40)	.04
		76th-100th percentile	0.0722 (0.26)	0.0733 (0.26)	0.0694 (0.25)	<.001

^a^Admission between 7 PM and 7 AM.

^b^SDoH: social determinants of health.

### Model Performance and Selection

Table 2 shows the algorithm performance measured on the test set for the 9 models run on 3 input combinations and 3 classifiers (logistic regression, RF, and GBM). ML classifiers consistently outperformed the benchmark logistic regression in all 3 input combinations. More specifically, the GBM classifier consistently outperformed logistic regression and RF in the first 2 input combinations (ie, when patient- and visit-level feature sets were used). When the patient-, visit-, and community-level variables were included as inputs (ie, the most complete input combination), ML models dominated the logistic regression. Inclusion of the individual-level SDoH features improved the performance for all 3 classifiers, especially the GBM model, where AUC increased from 0.694 (model 3) to 0.823 (model 6). Further inclusion of the community-level SDoH features improved the overall predictive performance measures, AUC, sensitivity, and specificity of the 2 ML models (models 8 and 9).

We based our model selection on both the performance metrics and the clinical needs in actual care settings. Note that the cost of misdiagnosis is asymmetrical. Misdiagnosis of a stroke (labeling a true stroke as a nonstroke condition) might have more severe adverse consequences for both patients and providers than overdiagnosis (ie, false-positive stroke diagnosis). Hence, the selected model should provide high sensitivity while maintaining specificity within a reasonable range. Both ML models (RF and GBM) correctly detected at least 97% (101,522/104,662) of all the patients that did have a stroke and thus significantly outperformed the prehospital stroke prediction scales (ranging between 0.38 and 0.62) [61] by a large margin. The Youden index, calculated by deducting 1 from the sum of the test’s sensitivity and specificity, was used to evaluate the overall discriminative power of the diagnostic test. The Youden index was not included in Table 2 because of space limitations; however, it can be easily calculated using sensitivity and specificity, both of which are included in the table. According to several recent literature reviews, the Youden index of the stroke prediction scales used in the emergency medical services, ambulances, and emergency room settings ranged from 0.30 to 0.54 [61,62], whereas that of our stroke prediction models ranged from 0.56 to 0.62.

Multimedia Appendix 4 presents the results of using the alternative data split method by using the historical 2012 data to train the model. Our model still demonstrated good overall performance with a high sensitivity rate of >90% and *F*_1_-score in the range of 0.83 to 0.88.

**Table 2:** Performance of stroke prediction models.

Input combinations and model number			Classifier		Accuracy		AUC^a^		Sensitivity		Specificity		*F*_1_-score
**Patient demographics and visit information**													
	1	Logit		0.828		0.693		0.960		0.626		0.893	
	2	RF^b^		0.804		0.680		0.928		*0.632* ^c^		0.877	
	3	GBM^d^		*0.832*		*0.694*		*0.968*		0.619		*0.896*	
**Patient demographics, visit information, and individual SDoH^e^**													
	4	Logit		0.830		0.810		0.960		0.630		0.895	
	5	RF		0.794		0.724		0.899		*0.656*		0.868	
	6	GBM		*0.835*		*0.823*		*0.965*		0.631		*0.898*	
**Patient demographics, visit information, individual SDoH, and community-level SDoH**													
	7	Logit		0.822		0.810		0.967		0.629		0.891	
	8	RF		0.831		0.828		*0.972*		0.626		0.896	
	9	GBM		*0.834*		*0.829*		0.970		*0.647*		*0.898*	

^a^AUC: area under the receiver operating characteristic curve.

^b^RF: random forest.

^c^For each input combination, the best performance among the 3 classifiers has been italicized.

^d^GBM: gradient boosting machine.

^e^SDoH: social determinants of health.

### Feature Importance Analysis

**Figure 3:**
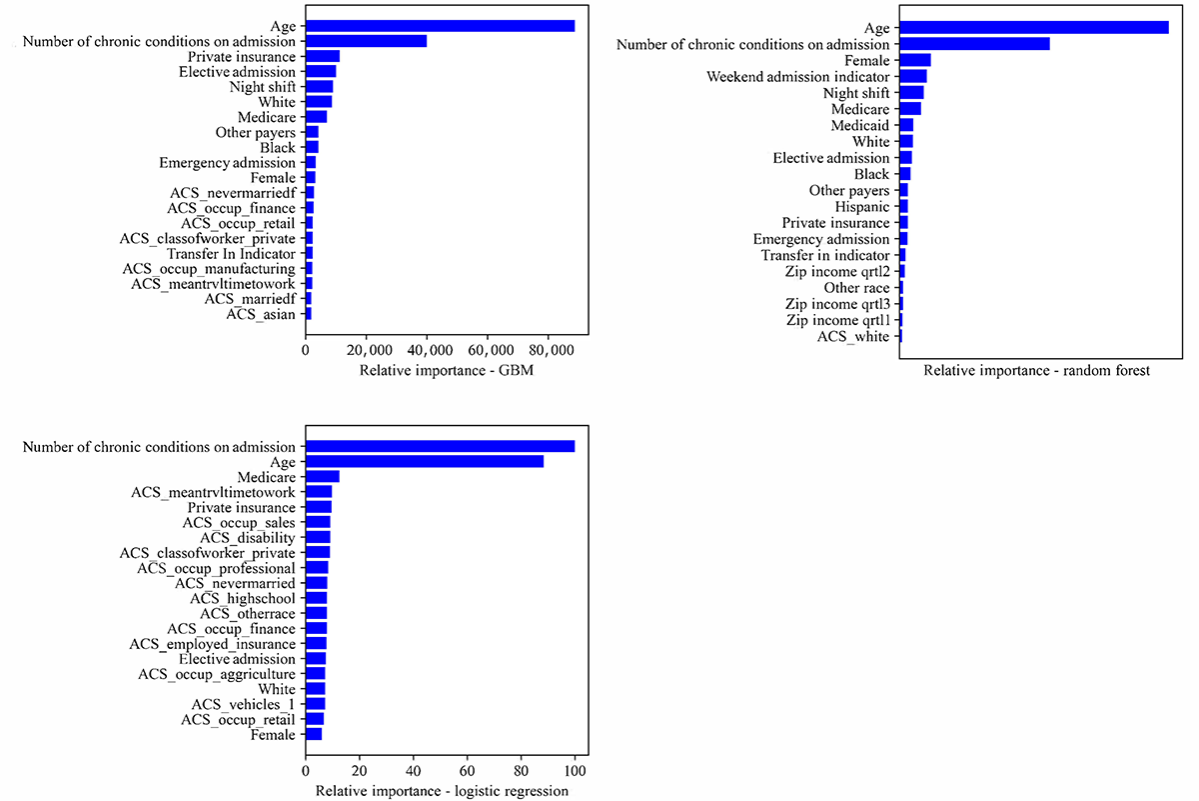
Comparison of feature importance: 20 most important features for gradient boosting machine (GBM; upper left), random forest (upper right), and logistic regression (bottom). ACS: American Community Survey; Qrtl: quartile.

We found consistency across the 3 models in the most important features that explain their performance (Figure 3; glossary of the terms used as well as the variable definitions can be found in Multimedia Appendix 5). The top 5 features deal with age, the number of chronic conditions on admission, and primary payer (eg, Medicare or private insurance). The top 2 features were age and the number of chronic conditions across all 3 models. Both the RF and GBM models identified patients’ age as one of the most important features. This is consistent with the findings of a recent study that used a new hybrid feature selection model that integrated various filter and wrapper methods to detect stroke risk [63]. Older age increases the predicted stroke probability, and younger age decreases the predicted probability. The second most important feature contributing to the models’ performance was the number of chronic conditions on admission. A higher number of chronic conditions on admission increases the predicted stroke probability.

It is interesting to note that the patients’ admission type (eg, whether it is an emergency or elective admission) and timing of admission (ie, whether they were admitted during the night shift) contributed to the accuracy of stroke prediction. Existing studies have investigated the presence of a “weekend effect” on mortality [64-66] and the differences in the quality of treatment that patients receive based on their time of hospital arrival or admission [67,68]. In general, these studies primarily focused on emergency admissions. If adverse patient outcomes such as mortality are related to different work practices and staff availability during off-hour periods, then the diagnosis of acute conditions is likely to be similarly affected, and our current findings confirm this hypothesis.

In addition to age, other patient-level demographic and socioeconomic factors, including gender, race, and primary payer (ie, whether the medical expenses were covered by Medicare, Medicaid, private insurance, or other payers), contribute to the models’ prediction. These findings complement the recently observed diverging stroke risk patterns among different racial and gender groups [69,70]. For instance, Howard et al [69] found that when aged between 45 and 74 years, White women were less likely to have a stroke than White men; however, there was no difference in stroke risk between White men and women when the latter were aged ≥75 years. By contrast, they found that Black women were at a lower risk of stroke than Black men when they were aged ≤64 years and experienced a similar stroke risk thereafter [69]. Another study found that Black women had a greater risk of stroke than White women, and the racial disparities were greatest among women aged 50 to 60 years [70]. In addition, our findings revealed that health insurance status is not only associated with health care use but also an important predictor of stroke. These findings have important implications and suggest that a cookie-cutter approach may not work well for stroke prevention. For instance, interventions targeting socially disadvantaged individuals without Medicare coverage may provide the greatest benefit in reducing disparities.

Some community-level SDoH variables (eg, percentage of single women; percentage of people with occupations closely related to finance, retail, and manufacturing industries; and mean travel time to work) were also among the top 20 features. However, the magnitude of their impact on stroke prediction was much less than that of patient-level demographic and socioeconomic features. This is consistent with the literature [40] and with the predictive performance reported in Table 2. Although both the individual- and community-level SDoH features helped improve the predictive performance, the inclusion of the individual-level SDoH features led to a much larger improvement (AUC increased from 0.694 to 0.823) than the inclusion of the community-level SDoH features (AUC increased from 0.823 to 0.829). Only adding the community-level SDoH features to the visit-level data (in the absence of individual-level SDoH features) increased the AUC from 0.694 to 0.724.

Ablation studies are commonly used for assigning importance scores to features [71-73]. In this method, the importance of a feature is decided based on the reduction in performance that its removal causes. We performed the ablation analysis as follows. First, we trained the GBM model on the training data set and calculated the base score on the testing data set using the accuracy metric. Second, we removed one feature from the training data set, trained the GBM model again, and then calculated the score of the model on the testing data set. This was repeated for each feature included in the model. Finally, we ranked the features based on the difference between the score calculated in their absence and the base score (calculated when all the features were present). Consistent with the findings from the feature importance comparison analysis, the top 2 highest-ranked features based on the ablation analysis were age and the number of chronic conditions, followed by the individual-level SDoH features and then the community-level SDoH features (Multimedia Appendix 6).

### Individual Prediction Explanations

To provide insights into prediction and ultimately assist care providers in decision-making, we sought to explain the stroke prediction model using TreeSHAP [74], a variant of Shapley Additive Explanations (SHAP) for tree-based ML models. The SHAP method computes Shapley values from the coalitional game theory to quantify the contributions of each feature to the prediction [75-77]. TreeSHAP uses conditional expectation to estimate the effects for a single tree, and the Shapley values of a tree ensemble are the weighted average of the Shapley values of the individual trees.

**Figure 4:**
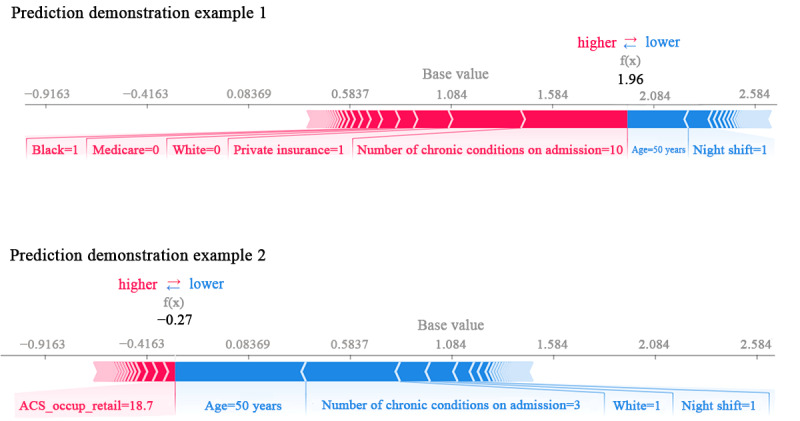
Shapley Additive Explanations values for example patients. ACS: American Community Survey.

Figure 4 shows SHAP values to explain the stroke prediction of 2 example cases (glossary of the terms used as well as the variable definitions can be found in Multimedia Appendix 5). We visualized feature attributions as “forces,” and each feature value is a force that either increases or decreases the prediction starting from the baseline. The base value or the expected value is the average of the model output over the training data and equals 1.084 [75]. Features that push the prediction higher (to the right) are shown in red, and those pushing the prediction lower are in blue. The first example (prediction demonstration example 1) obtained an output value (ie, prediction for this observation) of 1.96, higher than the base value and hence, this example was labeled by the prediction model as stroke. Being Black, having 10 chronic conditions on admission, and having private insurance as the primary payer pushed the stroke prediction higher. This is consistent with the literature suggesting that the odds of a probable misdiagnosis of a stroke in the EDs were lower among Medicare or Medicaid recipients than among privately insured patients [7]. In comparison, we also looked at the SHAP values for another example (prediction demonstration example 2), where the model successfully predicts a stroke mimic. This second example obtained a low output value of −0.27. Similar to demonstration example 1, demonstration example 2 was aged 50 years; however, being White person, having 3 chronic conditions on admission, and being admitted during a night shift pushed the stroke prediction lower.

These examples demonstrate that individual-level predictors of stroke can differ significantly from one case to another and can be used for personalized diagnostic and treatment decisions at the point of care, whereas the population-level analysis provides an overall ranking of the important predictors of stroke at hospital presentation and can be used to develop best practice guidelines and patient management programs.

## Discussion

### Principal Findings

In this study, we developed an ML-based approach using routinely collected administrative data to help reduce stroke misdiagnosis. Our findings suggest that before obtaining diagnostic imaging or laboratory test results, it is possible to predict stroke based on patients’ demographics and SDoH information available at the time of hospital presentation. The algorithm had an AUC of 83%, provided accurate results (high precision of 84%), and returned a supermajority (101,522/104,662, 97%) of all positive results (high sensitivity).

**Figure 5:**
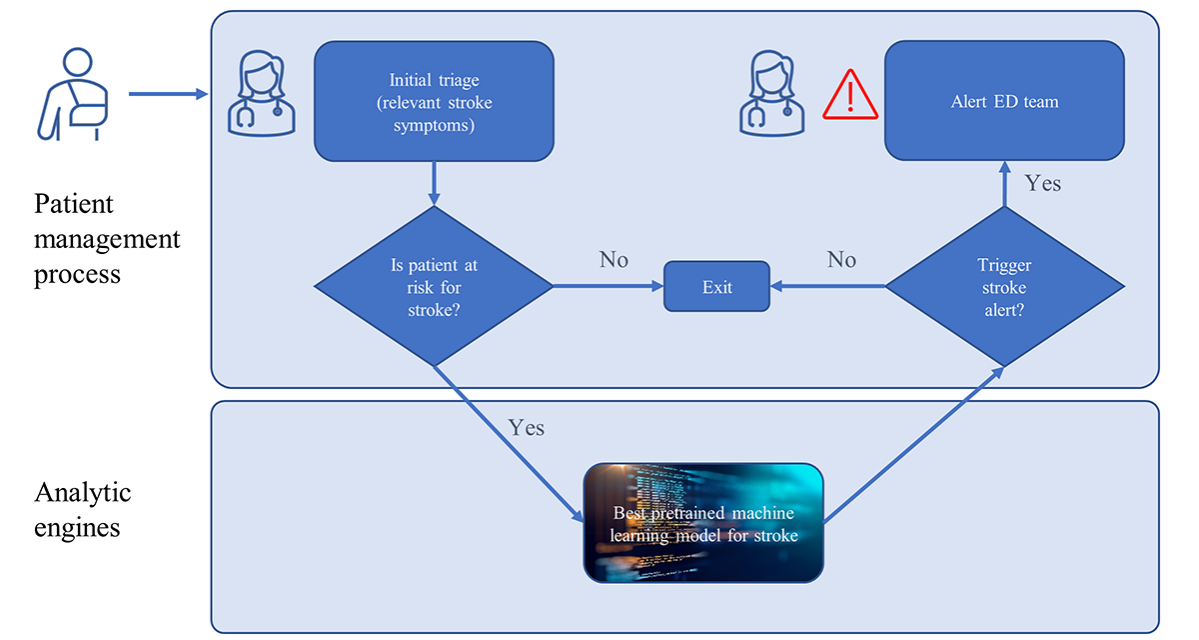
Decision support for stroke prediction. ED: emergency department.

This study fills a critical gap in the current efforts to support stroke triage, which either focuses on improving specificity in the prehospital setting or requires detailed neurological assessments and imaging results. On the one hand, advanced ML techniques have been applied to assist in automatically interpreting clinical notes and imaging, but this is based on the availability of these information sources. On the other hand, because emergency medical service personnel lack the necessary time and training to perform detailed neurological assessments, short and simple clinical methods known as prehospital stroke scales have been developed to support the initial triage in the field, such as the Cincinnati Prehospital Stroke Scale, Los Angeles Prehospital Stroke Scale, and Conveniently Grasped Field Assessment Stroke Triage. These scales have demonstrated wide performance variability in clinical practice; however, in general, they were found to have acceptable-to-good specificity but low sensitivity [62,78-80]. Literature reviews that compared studies with different prehospital stroke scales found that these scales varied in their accuracy and misdiagnosed up to 30% of acute strokes in the field. Depending on the sample and study site, Los Angeles Prehospital Stroke Scale and Cincinnati Prehospital Stroke Scale had similar diagnostic capabilities with sensitivity ranging from 0.79 to 0.91, and the sensitivity of using the Conveniently Grasped Field Assessment Stroke Triage in detecting large vessel occlusion stroke was 0.62. This means that these scales help detect false positives and thus reduce the wasteful use of medical resources. However, their low sensitivity has led to concerns that these scales will miss a substantial percentage of people with stroke. Hence, it is important to have an additional screening or decision support tool to supplement clinical assessment and provide valuable information to increase the sensitivity in detecting stroke at hospital presentation and thus reduce missed diagnoses [81]. In crowded hospitals, and with shortage of medical resources and clinical staff, the ML-based model we proposed can help quickly prioritize patients for appropriate intervention. If a patient presents with stroke or stroke-like symptoms, an automated, computer-assisted screening tool will be triggered to quickly analyze all the patient’s information available at the time of hospital presentation and suggest a diagnosis based on the best pretrained ML model for stroke. If the model predicts that the patient is at a high risk for stroke, a stroke pop-up will be triggered to alert the ED team. Figure 5 illustrates when and how this decision support prediction can be implemented in the field.

This model can be integrated with other AI-enabled prediction or decision support systems based on EHRs in the ED to further improve stroke triage and diagnosis. Although EHR data contain rich and detailed clinical information, certain social and behavioral determinants that can also be important risk factors (eg, race) are both poorly represented (including a category for “Unknown”) and inadequately characterized in the EHR [82]. Furthermore, various obstacles such as the lack of interoperability have limited the full use of EHR data to improve the delivery of care. Consequently, existing studies are mostly based on patient data confined to a single EHR system within a single geographic area [83]. By contrast, administrative data, such as claims data, follow specific standards for both the structure and meaning of the variables contained within a claim, and nearly every health care provider must submit electronic claims in the same format to their payers or clearinghouses. Hence, such administrative data provide an efficient way to complement EHR data in measuring many important aspects of health care delivery and provide solutions. We obtained the best of both worlds by leveraging the widely available administrative data with SDoH information to screen and quickly prioritize patients at hospital presentation and then using EHR data with rich clinical documentation and diagnostic test results to further assess and stratify patients based on risk.

### Comparison With Prior Work

It is important to consider specific clinical needs and care settings when comparing the various forms of performance measures reported across studies. In the case of strokes, misdiagnosis of a stroke (labeling a true stroke as a nonstroke condition) usually leads to more severe adverse patient outcomes than overdiagnosis. Although false-positive stroke mimics rarely lead to legal consequences, false negatives can cause delays in critical treatments and often give rise to accusations of medical errors. Moreover, given the inherent trade-off between sensitivity and specificity, the prehospital stroke scales’ focus on specificity (ie, reducing overdiagnosis) may result in a substantial number of misdiagnoses of strokes that need to be addressed at patients’ hospital presentations. Therefore, minimizing the false-negative rate or maximizing the sensitivity is paramount in acute care settings for both patients and providers. Several recent studies have compared the currently available clinical assessment tools such as the field stroke triage scale, National Institute of Health Stroke Scale, Los Angeles Motor Scale, and Rapid Arterial Occlusion Evaluation, which incorporate cortical signs (eg, gaze deviation, aphasia, and neglect) as well as motor dysfunction, and found that these tools had better diagnostic accuracy for detecting patients with large vessel occlusion than for distinguishing between acute stroke and stroke mimics [81]. Many studies were designed such that patients with hemorrhage or stroke-mimicking conditions were excluded [81]. Clinical assessment tools aimed at distinguishing between acute stroke and stroke mimics demonstrated modest diagnostic accuracy with low sensitivity, ranging from 38% to 62%, in the prehospital setting [61]. The stroke diagnostic tools designed for ED settings, such as the Recognition of Stroke in the Emergency Room scale and the FABS scoring system, are found to have a higher sensitivity than the prehospital scales (up to 93%), and they require clinical assessments from neurologists, brain CT findings, or additional clinical information, such as atrial fibrillation [81,84]. To our knowledge, the sensitivity of the algorithms in this study, without relying on the availability of additional clinical information or imaging findings, outperforms any scoring scales used in the prehospital or ED settings.

This study is also one of the first large-scale studies to systematically assess the added value of SDoH information in a population-based risk-prediction setting using administrative data. Although many studies have shown that various social or behavioral factors are associated with health outcomes, very few have explicitly examined whether the knowledge of these factors improves the prediction of clinical events or health outcomes. Our results are consistent with the findings of nascent studies that link SDoH data with EHR data to predict ED visits [85] or the need for various social service referrals [86]. However, because EHR systems have not achieved full interoperability yet, these studies are mostly confined to patient data from a single EHR system within a single geographic area. This study extends the literature by leveraging the routinely collected data that span different health care systems and regions to complement some of the necessary first steps associated with population health analytics. Moreover, the development of electronic health information exchanges helps bring together information from multiple sources and combine administrative claims data with clinical data. Such progress makes it possible to create an integrated profile of a patient at the time of hospital presentation and further empowers our predictive analytics*.*

### Limitations and Future Research

This study has room for further improvement, which is left for future research. First, this was a retrospective study, and confirmation of stroke cases relied on International Classification of Diseases codes. It is desirable to have patients’ complicated medical records reviewed to ascertain stroke diagnosis; however, this process is labor intensive and expensive, especially when it is a large-scale study with hundreds of thousands of patients across different health systems. Our results require further validation but have the potential for improving stroke triage and diagnosis.

Second, the algorithm we proposed should not be considered as the gold standard for stroke diagnosis. Rather, we believe that the algorithm complements the existing stroke scoring systems used in the prehospital or emergency room settings and can be integrated into ML-enabled decision support systems that combine patients’ medical history, SDoH, and clinical data. Such a decision system would have the advantage of being agile and iterative, in the sense that the model outcome can be reassessed at regular intervals as more data are collected in the ED, as well as the integration of variables with the most promising relevance.

Third, the focus of this study was to predict stroke solely based on the information available at the time of a patient’s initial presentation at the hospital. This is because first-time or new patients with stroke make up the supermajority (77%) of the yearly US patient population with stroke [87], and it is more challenging to make stroke predictions accurately for those patients who show up at the ED for the first time with no historical data. Patients with repeated readmissions and single-visit patients may follow different trajectories with different underlying social and behavioral determinants [88]. Future research may continue to explore how to best incorporate past information to improve prediction and identify key risk factors for repeated patients.

Finally, our findings are limited to the SDoH variables available in administrative data, suggesting the importance of developing standards and tools to routinely collect and screen individual-level SDoH data and effectively integrate them into both EHR and structured claims data. Our current prediction does not require any additional effort to collect additional individual-level SDoH. The community-level ACS variables have already been incorporated as part of the best pretrained model. The patient-level details used in our prediction are (1) basic demographics including age, gender, race and ethnicity, and primary payer (ie, Medicare, Medicaid, private insurance, or others); (2) arrival information (eg, whether it was an emergency or elective admission and whether the patient was admitted during a weekend or night shift); and (3) whether the patient resided in an urban or a rural area and the quartile in which their median household income fell (Table 1), both of which are based on the zip code variable. All 3 categories of information are routinely collected by triage nurses at hospital EDs during the initial triage. For instance, the zip code can be obtained from the patient’s home address, and the primary payer can be identified from the insurance information. Hence, patients do not need to answer any additional SDoH-related questions for the currently proposed prediction. To include more patient-level SDoH and further improve the predictive performance, more efforts are needed to develop SDoH screening and collection tools. National efforts are underway starting with primary care, such as the Protocol for Responding to and Assessing Patients' Assets, Risks, and Experiences survey, a standardized patient risk assessment tool consisting of a set of national core measures for addressing patients’ SDoH. Future research can also leverage more advanced ML algorithms (eg, deep learning) to facilitate a more comprehensive and efficient analysis of the large, high-dimensional data sets with claims, EHR, and SDoH data.

### Conclusions

Stroke is among the most common and dangerous misdiagnosed medical conditions. Black people, Hispanic people, women, older people on Medicare, and people in rural areas are less likely to be diagnosed in time for treatment after having a stroke. Timely detection is the key to effective management and improved patient outcomes.

We developed a high-performance ML-based stroke prediction algorithm that outperforms the existing early warning scoring systems. The algorithm is based on variables routinely collected and readily available at the time of patients’ hospital presentations and may provide an opportunity for enhanced patient monitoring and stroke triage and improved health outcomes. Because the prediction model does not require clinical notes or diagnostic test results, it can be particularly useful in underresourced EDs in rural and underserved communities with limited availability of sensitive diagnostic tools and incomplete data-gathering capabilities. Moreover, the algorithm can be incorporated into an automated, AI-enabled decision support system that combines administrative data widely available at the time of ED presentation and subsequently available clinical notes and diagnostic test results to further improve stroke diagnosis, triage, and management.

## Appendix

Multimedia Appendix 1 The top 20 principal diagnoses in the analysis sample.

Multimedia Appendix 2 Community-level social determinants of health variables.

Multimedia Appendix 3 Tuned hyperparameters in machine learning models.

Multimedia Appendix 4 Performance of the stroke prediction models based on the alternative data split method.

Multimedia Appendix 5 Glossary of the terms used as well as the variable definitions.

Multimedia Appendix 6 Results of the ablation analysis.

## Abbreviations

ACS: American Community Survey
AUC: area under the receiver operating characteristic curve
CT: computed tomography
ED: emergency department
EHR: electronic health record
GBM: gradient boosting machine
ML: machine learning
RF: random forest
SDoH: social determinants of health
SHAP: Shapley Additive Explanations
