# Supplementary material for: A Machine Learning Approach to Support Urgent Stroke Triage Using Administrative Data and Social Determinants of Health at Hospital Presentation: Retrospective Study
Source: J Med Internet Res. 2023 Jan 30;25:e36477. doi: 10.2196/36477 (PMC9926350; doi:10.2196/36477)
Supplement: Multimedia Appendix 1 [file jmir_v25i1e36477_app1.docx]

# Multimedia Appendix 1: The Top 20 Principal Diagnoses in the Analysis Sample

| ICD-9-CM Code | Principal Diagnosis | Frequency | Percentage |
| --- | --- | --- | --- |
| 43491 | Cerebral artery occlusion, unspecified with cerebral infarction | 63,485 | 44.33% |
| 43411 | Cerebral embolism with cerebral infarction | 11,819 | 8.25% |
| 431 | Intracerebral hemorrhage | 10,744 | 7.50% |
| 78039 | Other convulsions | 10,271 | 7.17% |
| 34590 | Epilepsy, unspecified, without mention of intractable epilepsy | 5,992 | 4.18% |
| 43311 | Occlusion and stenosis of carotid artery | 5,186 | 3.62% |
| 4321 | Subdural hemorrhage | 4,380 | 3.06% |
| 430 | Subarachnoid hemorrhage | 4,085 | 2.85% |
| 25080 | Diabetes with other specified manifestations, type II or unspecified type, not stated as uncontrolled | 2,107 | 1.47% |
| 43401 | Cerebral thrombosis with cerebral infarction | 1,707 | 1.19% |
| 4329 | Unspecified intracranial hemorrhage | 1,569 | 1.10% |
| 29181 | Alcohol withdrawal | 1,545 | 1.08% |
| 34510 | Generalized convulsive epilepsy, without mention of intractable epilepsy | 1,353 | 0.94% |
| 3453 | Grand mal status | 771 | 0.54% |
| 34580 | Other forms of epilepsy and recurrent seizures, without mention of intractable epilepsy | 757 | 0.53% |
| 1983 | Secondary malignant neoplasm of brain and spinal cord | 560 | 0.39% |
| 2512 | Hypoglycemia, unspecified | 542 | 0.38% |
| 43321 | Occlusion and stenosis of vertebral artery with cerebral infarction | 507 | 0.35% |
| 34550 | Localization-related (focal) (partial) epilepsy and epileptic syndromes with simple partial seizures, without mention of intractable epilepsy | 502 | 0.35% |
| 2920 | Drug withdrawal | 483 | 0.34% |
