# Supplementary material for: A Machine Learning Approach to Support Urgent Stroke Triage Using Administrative Data and Social Determinants of Health at Hospital Presentation: Retrospective Study
Source: J Med Internet Res. 2023 Jan 30;25:e36477. doi: 10.2196/36477 (PMC9926350; doi:10.2196/36477)
Supplement: Multimedia Appendix 2 [file jmir_v25i1e36477_app2.docx]

# Multimedia Appendix 2: Community-level SDoH Variables

| Category of Features | Number of Variables | Descriptions |
| --- | --- | --- |
| **I. Area Basic Demographics** | | |
| Living situation | 10 | % of households with female head of household; % of households with householder living alone; avg household size; % of population living in the same house as 1 year ago; % of housing units that are mobile homes; % of housing units with no vehicle, 1 vehicle, 2 vehicles, or 3+ vehicles; % of housing units with no telephone |
| Race | 7 | % of population with race white, race black, race Native American, race Asian, or other race; % of population with 2 or more races; % of population Hispanic |
| Marital status | 6 | % of males never married, married, or divorced; % of females never married, married, or divorced |
| Housing cost | 3 | median monthly owner housing costs with mortgage; median monthly owner housing costs without mortgage; median monthly rent for rented units |
| Language | 3 | % of population with language spoken at home being English only, non-English, or English spoken less than well |
| Disability | 2 | % of population with disability; % of population over 65 with disability |
| Fertility | 1 | % of women who had a birth in the past 1 year |
| Veteran status | 1 | % of population who are veterans |
| Gini | 1 | Gini index of inequality |
|  |  |  |
| **II. Socio-Economic Status** | | |
| Poverty | 4 | % of families below the poverty level; % of families with children below the poverty level; % of families with female head of household below the poverty level; % of families with female head of household and children below poverty level |
| Income/Wealth | 2 | median household income; median value of owner's unit |
| Education | 2 | % of population with at least high school degree, or with at least bachelor’s degree |
|  |  |  |
| **III. Occupation** | | |
| Occupation | 23 | % of workers employed in management, service, sales, natural resources, transportation, agriculture, construction, manufacturing, wholesale, finance, professional services, education, arts, public administration, other; % of private workers; % of government workers; % of self-employed workers; % of unpaid workers; % of the labor force who are unemployed |
| Commute to work | 7 | % of workers whose commute method is driving, carpooling, public transportation, walking, or other; % of workers who work at home; average travel time to work in minutes |
|  | | |
| **IV. Health Insurance** | | |
| Health insurance coverage | 6 | % of population with private health insurance, public health insurance, or no health insurance; % of population under 18 with no health insurance; % of the employed population with health insurance, and with no health insurance |
|  | | |
