# Supplementary material for: A Machine Learning Approach to Support Urgent Stroke Triage Using Administrative Data and Social Determinants of Health at Hospital Presentation: Retrospective Study
Source: J Med Internet Res. 2023 Jan 30;25:e36477. doi: 10.2196/36477 (PMC9926350; doi:10.2196/36477)
Supplement: Multimedia Appendix 3 [file jmir_v25i1e36477_app3.docx]

# Multimedia Appendix 3: Tuned Hyper-parameters in Machine-learning Algorithms

| Machine learning algorithm | Hyper-parameter | Description | Value |
| --- | --- | --- | --- |
| Logistic regression^^[[1]](#footnote-1)^^ | Penalty | Norm used in the penalization | None |
|  | C | Inverse of regularization strength | 1.0 |
|  | tol | Tolerance for stopping criteria | 0.0001 |
|  | class_weight | Weights associated with classes | Balanced |
| Random Forest^^[[2]](#footnote-2)^^ | n_estimators | Number of trees in the forest | 300 |
|  | max_depth | Maximum depth of the tree | 16 |
|  | min_samples_split | Minimum number of samples required to split an internal node | 10 |
|  | max_features | Number of features to consider when looking for the best split | 20 |
|  | class_weight | Weights associated with classes | Balanced |
| Gradient Boosting Machine^^[[3]](#footnote-3)^^ | learning_rate | Used for reducing the gradient step | 0.1 |
|  | num_trees | The maximum number of trees that can be built when solving machine learning problems | 500 |
|  | depth | Depth of the tree | 3 |
|  | auto_class_weights | Automatically calculate class weights based either on the total weight or the total number of objects in each class. The values are used as multipliers for the object weights | Balanced |

1. Implemented in in Python 3.9.12 using scikit-learn (version 1.0.2) [↑](#footnote-ref-1)
2. Implemented in in Python 3.9.12 using scikit-learn (version 1.0.2) [↑](#footnote-ref-2)
3. Implemented in in Python 3.9.12 using catboost (version 1.0.6) [↑](#footnote-ref-3)
