# Supplementary material for: A Machine Learning Approach to Support Urgent Stroke Triage Using Administrative Data and Social Determinants of Health at Hospital Presentation: Retrospective Study
Source: J Med Internet Res. 2023 Jan 30;25:e36477. doi: 10.2196/36477 (PMC9926350; doi:10.2196/36477)
Supplement: Multimedia Appendix 4 [file jmir_v25i1e36477_app4.docx]

**Multimedia Appendix 4: Performance of the Stroke Prediction Models based on Alternative Data Split Method**

| Alternative Data Split Method | Classifier | Accuracy | Precision | Specificity | Sensitivity | F1 Score |
| --- | --- | --- | --- | --- | --- | --- |
| Using 2012 to Predict 2013 | Logistic Regression | 0.799 | 0.841 | 0.586 | 0.895 | 0.867 |
|  | Random Forest | 0.797 | 0.838 | 0.576 | 0.895 | 0.866 |
|  | Gradient Boosting Machine | 0.809 | 0.842 | 0.579 | 0.911 | 0.832 |
| Using 2012 and 2013 to Predict 2014 | Logistic Regression | 0.799 | 0.841 | 0.574 | 0.897 | 0.868 |
|  | Random Forest | 0.804 | 0.842 | 0.572 | 0.904 | 0.872 |
|  | Gradient Boosting Machine | 0.811 | 0.843 | 0.579 | 0.914 | 0.877 |

Note: Input combination = Patient demographics and Basic Visit Information + Individual Level SDoH + ACS Community-level SDoH
