# Supplementary material for: A Machine Learning Approach to Support Urgent Stroke Triage Using Administrative Data and Social Determinants of Health at Hospital Presentation: Retrospective Study
Source: J Med Internet Res. 2023 Jan 30;25:e36477. doi: 10.2196/36477 (PMC9926350; doi:10.2196/36477)
Supplement: Multimedia Appendix 5 [file jmir_v25i1e36477_app5.docx]

# Multimedia Appendix 6: Glossary of Terms for Figures 3 and 4

| Abbreviation | Definition |
| --- | --- |
| ACS_nevermarriedf | The percentage of females living in a specific zip code never married. |
| ACS_occup_finance | The percentage of people living in a specific zip code who had occupations closely related to the finance industry. |
| ACS_occup_retail | The percentage of people living in a specific zip code who had occupations closely related to the retail industry. |
| ACS_classofworker_private | The percentage of people living in a specific zip code who had the type of ownership of the employing organization in the private sector. |
| ACS_occup_manufacturing | The percentage of people living in a specific zip code who had occupations closely related to the manufacturing industry. |
| ACS_meantrvtimetowork | Mean travel time from home to work each day in minutes. |
| ACS_marriedf | The percentage of females living in a specific zip code who got married. |
| ACS_asian | The percentage of people living in a specific zip code having origins in any of the original peoples of the Far East, Southeast Asia, or the Indian subcontinent including, for example, Cambodia, China, India, Japan, Korea, Malaysia, Pakistan, the Philippine Islands, Thailand, and Vietnam. |
