# Supplementary material for: A Machine Learning Approach to Support Urgent Stroke Triage Using Administrative Data and Social Determinants of Health at Hospital Presentation: Retrospective Study
Source: J Med Internet Res. 2023 Jan 30;25:e36477. doi: 10.2196/36477 (PMC9926350; doi:10.2196/36477)
Supplement: Multimedia Appendix 6 [file jmir_v25i1e36477_app6.docx]

# Multimedia Appendix 5: Results of the Ablation Analysis

| Feature | Difference with base score |
| --- | --- |
| Age | 0.03383 |
| Number of Chronic Conditions on Admission | 0.00406 |
| White | 0.00070 |
| Transfer In Indicator | 0.00040 |
| Medicare as the Primary Payer | 0.00029 |
| % of Population under 18 with no Health Insurance | 0.00026 |
| % of Workers employed in Transportation | 0.00022 |
| % of Housing Units with No Vehicle | 0.00022 |
| % of Women Who Had a Birth in the Past 1 year | 0.00011 |
| Hispanic | 0.00004 |
| Median Household Income for the Patient's ZIP Code of Residence in Quartile 1^[[1]](#footnote-1)^ | 0.00004 |
| Median Household Income for the Patient's ZIP Code of Residence in Quartile 2^[[2]](#footnote-2)^ | 0.00004 |
| Logarithm of the % of Population with Disability | 0.00004 |
| % of Population with Language Spoken at Home being Non-English | 0.00004 |
| % of the Labor Force who are Unemployed | 0.00004 |
| % of Population with Occupation in Transportation and Warehousing, and Utilities | 0.00004 |
| % of Population with Occupation in Information | 0.00004 |
| % of Workers Works in the Private Sector | 0.00004 |
| % of Families and People Whose Income in the Past 12 Months is below the Poverty Level - All families with related children under 18 years | 0.00004 |

1. The first or bottom quartile of the median household income for the patient's ZIP code of residence is $1-$38,999 in 2012, $1-$37,999 in 2013, and $1-$39,999 in 2014. [↑](#footnote-ref-1)
2. The second quartile of the median household income for the patient's ZIP code of residence is $39,000-$47,9999 in 2012, $38,000-$47,999 in 2013, and $40,000-$50,999 in 2014. [↑](#footnote-ref-2)
